# Beta-adrenergic receptor blockade in angiosarcoma: Which beta-blocker to choose?

**DOI:** 10.3389/fonc.2022.940582

**Published:** 2022-09-15

**Authors:** Alaa Embaby, Lisanne van Merendonk, Neeltje Steeghs, Jos Beijnen, Alwin Huitema

**Affiliations:** ^1^ Department of Medical Oncology and Clinical Pharmacology, The Netherlands Cancer Institute-Antoni van Leeuwenhoek, Amsterdam, Netherlands; ^2^ Department of Pharmacy and Pharmacology, The Netherlands Cancer Institute-Antoni van Leeuwenhoek, Amsterdam, Netherlands; ^3^ Department of Pharmacology, Princess Máxima Center for Pediatric Oncology, Utrecht, Netherlands; ^4^ Department of Clinical Pharmacy, University Medical Center Utrecht, Utrecht University, Utrecht, Netherlands

**Keywords:** beta-blockade, angiosarcoma, drug repurposing, pharmacological characteristics, propranolol

## Abstract

Beta-blockers are currently studied to improve therapeutic options for patients with angiosarcoma. However, most of these patients have no cardiovascular co-morbidity and it is therefore crucial to discuss the most optimal pharmacological properties of beta-blockers for this population. To maximize the possible effectiveness in angiosarcoma, the use of a non-selective beta-blocker is preferred based on *in vitro* data. To minimize the risk of cardiovascular adverse events a beta-blocker should ideally have intrinsic sympathomimetic activity or vasodilator effects, e.g. labetalol, pindolol or carvedilol. However, except for one case of carvedilol, only efficacy data of propranolol is available. In potential follow-up studies labetalol, pindolol or carvedilol can be considered to reduce the risk of cardiovascular adverse events.

## Graphical Abstract

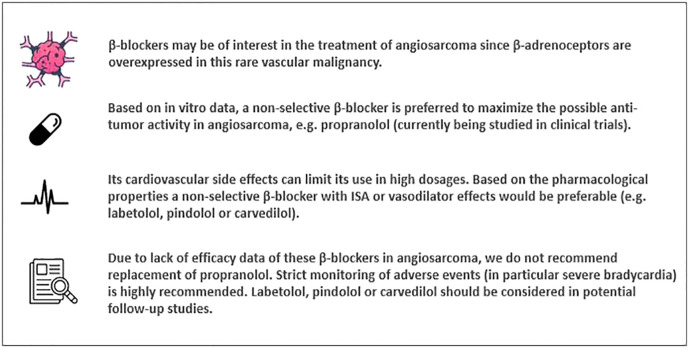

## Introduction

Beta (β)-blockers or β-adrenoceptor antagonists were first introduced in the early 1960s for the treatment of angina pectoris (1). The use of β-blockers in ischemic cardiac disease is based on the reduction of the catecholaminergic stimulation of the heart in order to improve the exercise capacity of the cardiac muscle by slowing heart rate and lowering the oxygen consumption. The anti-hypertensive and anti-arrhythmic effects of this drug class have led to its extensive use in the treatment of hypertension and cardiac arrhythmias. Other (off-label) indications for the use of β-blockers are migraine, the treatment of essential tremor, prophylaxis of esophageal variceal hemorrhage caused by portal hypertension, control of anxiety symptoms (exam anxiety and stage fright) and thyrotoxicosis.

The expression of β-adrenergic receptors has been investigated previously in both benign and malignant vascular tumors (2, 3). The registered indication of propranolol, a non-selective β-blocker, has now been extended to the treatment of infantile hemangioma requiring systemic therapy (4, 5). Since β-adrenoceptors are also overexpressed in some cancer types, such as soft tissue sarcomas, propranolol might be a drug of interest in these rare malignancies. Hence, propranolol obtained the orphan designation by the European Commission for the treatment of soft tissue sarcomas (6, 7). Furthermore, the effect of β-receptor blockade with propranolol on angiosarcoma is currently being studied in two clinical trials: The PropAngio trial (NCT04518124, study start date December 2019, status ongoing) and the PROPAN trial (NCT02732678, study start date May 2016, status unknown) (8–10). The aim of the PropAngio trial is to prospectively assess the anti-proliferative effect of neoadjuvant propranolol as monotherapy in primary, recurrent and metastatic angiosarcoma. In the PROPAN trial, propranolol is given in combination with cyclophosphamide in locally advanced or metastatic angiosarcoma (10). The usual daily dose range of propranolol for hypertension is 160 to 320 mg (5). In the PropAngio trial, propranolol is given orally in a dose-escalation schedule (40-80 mg twice daily in the first two weeks, thereafter 80 mg three times a day) until initiation of standard therapy (chemotherapy and/or surgery or radiation). Since the anti-proliferative effect of propranolol on angiosarcoma cells has been shown to be dose dependent, evaluating the effect of dose escalation in this trial is necessary (3). However, these patients have no cardiovascular co-morbidity or other indications for β-receptor blockade, making them more at risk for treatment related cardiovascular adverse events, mainly bradycardia and hypotension. These known side effects of propranolol can limit its use in high dosages in this patient population. Ideally, one should adapt a β-blocker with proven efficacy in angiosarcoma and which is characterized by minimal cardiovascular side effects. Here, we discuss the pharmacological properties of the diverse β-blockers and consider the usage of propranolol in angiosarcoma.

## Pharmacological properties

β-blockers can be distinguished based on their pharmacological properties by selectivity, intrinsic sympathomimetic activity (ISA), vasodilator activity and lipophilicity (**Table 1**). These pharmacological properties can be of use to define treatment and to predict adverse events. Intrinsic sympathomimetic activity or partial agonist activity of β-blockers is caused by inhibition of β-receptors in the presence of agonists, such as catecholamines, and stimulation of β-receptors in rest when these agonists are absent (11). This results in less bradycardia or peripheral vasoconstriction (12). Consequently, β-blockers with ISA can be of use in patients in whom strong decline of heart rate is unwanted. A comparison between pindolol and propranolol confirms this clinical benefit of β-blockers with ISA in patients with angina pectoris (13). Of the non-selective β-blockers with a vasodilator effect, there is some evidence that carvedilol causes less bradycardia compared to other β-blockers (14). Lipophilic β-blockers can more easily cross the blood-brain barrier increasing theoretically the incidence of central nervous system adverse events (12). However, studies were unable to associate lipophilicity to the incidence of adverse events (18).

**Table 1:** Properties of β-blockers divided into selectivity, intrinsic sympathomimetic activity and vasodilator activity (11–17).

	Group	Examples	Mechanism	Clinical impact
**Selectivity** (11, 12, 15, 16)	Selective β-blockers or cardioselective β-blockers	Acebutolol, atenolol, bisoprolol, celiprolol, esmolol, metoprolol, nebivolol	Higher affinity for β_1_ compared to β_2_. Selectivity can be lost at higher dosage	Less pulmonaric and metabolic adverse events**
	Non-selective β-blockers	Carvedilol, labetalol, pindolol, propranolol, sotalol	No selectivity for β_1_ or β_2_	N.A.
**Intrinsic sympathomimetic activity (ISA)** (11–13, 16)	β-blockers with ISA	Acebutolol, celiprolol, labetalol, pindolol*	At a low sympathetic tone stimulation of β-receptors	At rest less decrease in heart rate and cardiac output
	β-blockers without ISA	Atenolol, bisoprolol, carvedilol, esmolol, metoprolol, nebivolol, propranolol, sotalol	No stimulation of β-receptors	N.A.
**Vasodilator activity** (11, 12, 14, 16, 17)	Vasodilator activity	Carvedilol, labetalol, nebivolol	Carvedilol + labetalol: α_1_-receptor antagonismNebivolol: Nitric oxide (NO) release	Reduced peripheral vascular resistance
	No vasodilator activity	Acebutolol, atenolol, bisoprolol, celiprolol, esmolol, metoprolol, pindolol, propranolol, sotalolol	N.A.	N.A.
**Lipophilicity** (12, 15, 16)	Lipophilic	Acebutolol, bisoprolol, carvedilol, metoprolol, nebivolol, pindolol, propranolol	Passage of blood-brain barrier	Possible more central adverse events ***
	Hydrophilic	Atenolol, celiprolol, esmolol, labetalol, sotalol	Less passage of blood brain barrier	N.A.

*Most ISA compared to other β-blockers.

**β-blockers increase LDL and triglyceride levels and reduce HDL levels (more pronounced effect in non-selective β-blockers without ISA).

***e.g. sleep disturbances, psychosis, depression, hallucination.

β_1_-receptors are primarily located in the heart and most adverse effects of β-blockers are a result of antagonism of β_1_-receptors (notably bradycardia), whereas inhibition of β_2_-receptors, located on vascular and bronchial smooth muscle, can lead to adverse events like constriction of airways or peripheral vasculature (11, 15, 16). Therefore, cardioselective β-blockers are better tolerated and are preferred in patients with respiratory disease (15).

Differences in pharmacological properties can theoretically lead to varying adverse events. However, in head-to-head trials, the incidence of adverse events could not be clearly differentiated among most β-blockers (18, 19). Some studies report a difference in adverse events e.g.; carvedilol showed a higher rate of dizziness compared to metoprolol and propranolol was associated with a higher overall rate of adverse events compared to pindolol (13, 17). However, no β-blocker can be distinguished with highest incidence of adverse events (19).

## Use of β-blockers in angiosarcoma

Overexpression of β1-, β2- and β3-adrenoceptors in angiosarcoma cells has been demonstrated in several preclinical studies (2, 3). This finding suggests that, as in infantile hemangioma, β-blockers may also be of use in the treatment of this aggressive vascular tumor. Several mechanisms behind this anticancer effect have been suggested. Direct effects of β-blockers on tumor cells due to antagonism of β-receptors could lead to less proliferation, migration and differentiation of tumor cells (20). In addition, β-blockers can also influence tumor angiogenesis by downregulation of VEGF (2, 21–23). Furthermore, β-receptors play a role in immunostimulation and β-blockers can have effects on several immune cells (in both the adaptive and innate immune system) and on the tumor microenvironment (20, 22, 23). Several preclinical studies describe the synergy between β-blockers and chemotherapy resulting in less drug-resistance (3, 6, 21–24). Affecting the tumor microenvironment and immune system could also increase sensibility to immune checkpoint inhibitors by increasing T-cell infiltration and decreasing suppression of CD8+ cytotoxic T-cells (20, 25). Additionally the used isomer of β-blockers can be of importance. Studies on the isomers of propranolol suggest that the use of the R-isomer, which has no significant activity against the β-receptor, could lead to less side effects while maintaining efficacy. This supports a mechanism of action independent of β-blockade (24, 26). Using *in vitro* angiosarcoma models, it was found that the anti-proliferative effect of non-selective β-blockers was superior to cardioselective β-blockers (esmolol and atenolol) (27).

The anti-tumor activity of propranolol (as single agent and in combination with chemotherapy) in different types of angiosarcoma has been described in several case reports and case series (23, 28–35). **Table 2** shows the characteristics and results of these reports. Pasquier et al. reported the results of an unpowered pilot study in 7 patients with metastatic angiosarcoma in which propranolol was administered in combination with metronomic vinblastine-based chemotherapy. A response rate of 100% was observed (16). One single arm prospective clinical trial studied the efficacy of beta-blockade with propranolol or carvedilol in metastatic angiosarcoma (27). A total of 9 patients received either propranolol 20-100 mg per day (n=8) or carvedilol 6.25 mg per day (n=1) in combination with standard treatment protocols according to physicians choice. An improvement of the median progression-free survival (PFS) and overall survival (OS) was observed compared with historical controls (PFS 9 months vs. 3-6 months, OS 36 months vs. 12 months) (27).

**Table 2:** Case reports and case series of propranolol in angiosarcoma.

Report reference	Patient characteristics	Treatment given	Efficacy/best response	Toxicity
**Luczynska et al.** (28)	Female, 45 years, primary bilateral angiosarcoma of the breast	Doxorubicin + cyclophosphamide + propranolol 10 mg TID followed by surgery	Partial response (PR)	Not reported
**Fiste et al.** (29)	Male, 33 years, metastatic cardiac angiosarcoma	Paclitaxel (150 mg/m^2^)+ propranolol 40 mg TID	Progression-free survival (PFS) of 8 months	Manageable toxicity; not specified
**Daguze et al. 2017** (30)	Male, 49 years, nose angiosarcoma	Cyclophosphamide 200 mg QD (1 out of 2 weeks) + propranolol 120 mg daily	Complete remission after 2 months	Good tolerance; not specified
**Daguze et al. 2016** (31)	Male, 73 years, metastatic angiosarcoma of the scalp	Cyclophosphamide 200 mg QD (1 out of 2 weeks) + propranolol 120 mg daily	PR of visceral metastasis and CR of cutaneous lesions (minimal duration of response 7 months)	No severe toxicity
**Banavali et al.** (32)	Female, 69 years, metastatic angiosarcoma of left arm	Celecoxib 200 mg BID + etoposide 50 mg (for 3 months) + cyclophosphamide 50 mg daily during 15-21 days of a 28-day cycle + propranolol 40 mg BID	Complete response (CR). Relapse after 20 months from start of treatment.	No grade 3 or 4 toxicities
**Pramanik et al.** (33)	Female, 37 years, metastatic angiosarcoma of the breast	Oral thalidomide 200 mg daily + celecoxib 400 mg BID + intermittent etoposide 50 mg daily + intermittent cyclophosphamide 100 mg daily + propranolol 40 mg BID	SD	Neutropenia due to chemotherapy
**Galvan et al.** (34)	Female, 61 years, metastatic cardiac angiosarcoma	Propranolol monotherapy 40 mg/kg	On 12-month evaluation PET/CT regression of lesions and resolution of pericard effusion	Not reported
**Chow et al.** (35)	Male, 60+ years, angiosarcoma of the scalp	Neoadjuvant propranolol monotherapy 40 mg BID – 40 mg TIDFollowed by weekly paclitaxel (10 cycles) + daily propranolol. Thereafter combined with radiotherapy	Clinical improvement of lesion and decrease of proliferative index (also observed during propranolol monotherapy)	No adverse events observed (monotherapy)
**Pasquier et al.** (23)	7 patients (2 female, 5 male, age 20-72 years) with metastatic angiosarcoma	Metronomic vinblastine 6mg/m^2^ + methotrexate 35 mg/m^2^ + propranolol 40 mg BID. After 12 months intermittent cyclophosphamide + etoposide 50 mg daily + propranolol	100% response rate (very good partial response in n=3 and complete response in n=1).	Grade II fatigue in all patients

## Discussion

The aim of this perspective was to give an overview of the efficacy and safety of different β-blockers in angiosarcoma in order to recommend the use of the most appropriate β-blocker in this patient population. In accordance with the summarized preclinical and clinical data, we hypothesize that use of a non-selective β-blocker is preferred to maximize the possible effectiveness in angiosarcoma. Propranolol has been reported to be effective as monotherapy and in combination with chemotherapy in diverse cases of angiosarcoma. This is possibly the result of a synergistic effect of this non-selective β-blocker with chemotherapy by decreasing drug-resistance of tumor cells. β-blockade is also expected to enhance the immunomodulatory effects combinations of checkpoint inhibitors due to their positive effects on T-cells. However, propranolol is known to cause (cardiovascular) side effects by β-receptor antagonism, repurposing the R-isomer of propranolol could be a strategy to decrease side effects.

Given the abovementioned pharmacological features of β-blockers, one should ideally adopt a non-selective β-blocker with ISA to lower the risk of potential severe cardiovascular events such as bradycardia in patients with angiosarcoma. β-blockers that fulfill these criteria are labetalol and pindolol. As mentioned earlier, carvedilol has no ISA, but is known to cause less bradycardia because of its mainly vasodilator effects and may, therefore, be a suitable drug. To our knowledge, these agents have never been studied in angiosarcoma (except of carvedilol which is described in one case). Therefore, we do not recommend replacement of propranolol with another β-adrenoceptor antagonist in angiosarcoma due to lack of efficacy data. Hence, strict monitoring of adverse events and in particular (severe) bradycardia is highly recommended. The use of non-selective β-blockers with ISA should be considered in potential follow-up studies.

## Data availability statement

The original contributions presented in the study are included in the article/supplementary material. Further inquiries can be directed to the corresponding author.

## Author contributions

Conception and design: AE, LM, AH, and NS; Literature search and collection of data: AE and LM; Data interpretation: AE and LM; Manuscript writing: All authors. All authors contributed to the article and approved the submitted version.

## Conflict of interest

The authors declare that the research was conducted in the absence of any commercial or financial relationships that could be construed as a potential conflict of interest.

## Publisher’s note

All claims expressed in this article are solely those of the authors and do not necessarily represent those of their affiliated organizations, or those of the publisher, the editors and the reviewers. Any product that may be evaluated in this article, or claim that may be made by its manufacturer, is not guaranteed or endorsed by the publisher.

## References

[1] Baker JG Hill SJ Summers RJ. Evolution of β-blockers: from anti-anginal drugs to ligand-directed signalling. Trends Pharmacol Sci (2011) 32(4):227–34. doi: 10.1016/j.tips.2011.02.010 PMC3081074 21429598

[2] Chisholm KM Chang KW Truong MT Kwok S West RB Heerema-Mckenney AE. β-adrenergic receptor expression in vascular tumors. Modern Pathol (2012) 25(11):1446–51. doi: 10.1038/modpathol.2012.108 22743651

[3] Stiles JM Amaya C Rains S Diaz D Pham R Battiste J. Targeting of beta adrenergic receptors results in therapeutic efficacy against models of hemangioendothelioma and angiosarcoma. PloS One (2013) 8(3):e60021. doi: 10.1371/journal.pone.0060021 23555867 PMC3610939

[4] Léauté-Labrèze C Hoeger P Mazereeuw-Hautier J Guibaud L Baselga E Posiunas G. A randomized, controlled trial of oral propranolol in infantile hemangioma. N Engl J Med (2015) 372(8):735–46. doi: 10.1056/NEJMoa1404710 25693013

[5] Propranolol 10mg tablets BP - summary of product characteristics. Available at: https://www.medicines.org.uk/emc/product/5888/smpc#MACHINEOPS.

[6] Porcelli L Garofoli M di Fonte R Fucci L Volpicella M Strippoli S. The β-adrenergic receptor antagonist propranolol offsets resistance mechanisms to chemotherapeutics in diverse sarcoma subtypes: a pilot study. Sci Rep (2020) 10(1):10465. doi: 10.1038/s41598-020-67342-6 32591592 PMC7320177

[7] Medicines Agency E Public summary of opinion on orphan designation propranolol for the treatment of soft tissue sarcoma. Available at: www.ema.europa.eu/contact.

[8] Heinhuis KM Ijzerman NS Koenen AM van der Graaf WTA Haas RL Beijnen JH. PropAngio study protocol: a neoadjuvant trial on the efficacy of propranolol monotherapy in cutaneous angiosarcoma-a proof of principle study. BMJ Open (2020) 10(9). doi: 10.1136/bmjopen-2020-039449 PMC7485254 32912994

[9] Propranolol in angiosarcoma. Available at: https://clinicaltrials.gov/ct2/show/NCT04518124?term=propranolol&cond=Angiosarcoma&draw=2&rank=1.

[10] Dose-finding of propranolol in combination with metronomic fixed oral cyclophosphamide based on bivariate efficacy-tolerability outcome in patients with locally advanced or metastatic angiosarcoma: A collaborative and innovative phase I-II sequential trial by the French sarcoma group (GSF/GETO). Available at: https://clinicaltrials.gov/ct2/show/study/NCT02732678.

[11] Oliver E Mayor F Jr D’Ocon P. Beta-blockers: Historical perspective and mechanisms of action. Rev espanola cardiol (English ed) (2019) 72(10):853–62. doi: 10.1016/j.recesp.2019.02.023 31178382

[12] Poirier L Tobe SW. Contemporary use of β-blockers: clinical relevance of subclassification. Can J Cardiol (2014) 30(5 Suppl):S9–15. doi: 10.1016/j.cjca.2013.12.001 24684855

[13] Frishman W Kostis J Strom J Hossler M Elkayam U Goldner S. Clinical pharmacology of the new beta-adrenergic blocking drugs. part 6. a comparison of pindolol and propranolol in treatment of patients with angina pectoris. the role of intrinsic sympathomimetic activity. Am Heart J (1979) 98(4):526–35. doi: 10.1016/0002-8703(79)90261-8 39447

[14] Pedersen ME Cockcroft JR. The vasodilatory beta-blockers. Curr Hypertens Rep (2007) 9(4):269–77. doi: 10.1007/s11906-007-0050-2 17686376

[15] Farzam K Jan A. Beta Blockers. In: StatPearls [Internet]. Treasure Island (FL): StatPearls Publishing (2022).

[16] Borchard U. Pharmacological properties of β-adrenoceptor blocking drugs. J Clin Bas Cardiol (1998) 1(1):5–9.

[17] Metra M Giubbini R Nodari S Boldi E Modena MG Cas LD. Differential effects of beta-blockers in patients with heart failure: A prospective, randomized, double-blind comparison of the long-term effects of metoprolol versus carvedilol. Circulation (2000) 102(5):546–51. doi: 10.1161/01.CIR.102.5.546 10920067

[18] Ko DT Hebert PR Coffey CS Sedrakyan A Curtis JP Krumholz HM. Beta-blocker therapy and symptoms of depression, fatigue, and sexual dysfunction. JAMA (2002) 288(3):351–7. doi: 10.1001/jama.288.3.351 12117400

[19] Helfand M Peterson K Christensen V Dana T Thakurata S. Drug class review: Beta adrenergic blockers. Oregon Health and Science University (2009) p. 1–616. 21089245

[20] Wagner MJ Cranmer LD Loggers ET Pollack SM. Propranolol for the treatment of vascular sarcomas 2018. J Exp Pharmacol (2018) 10:51. doi: 10.2147/JEP.S146211 30233257 PMC6130307

[21] Pasquier E Ciccolini J Carre M Giacometti S Fanciullino R Pouchy C. Propranolol potentiates the anti-angiogenic effects and anti-tumor efficacy of chemotherapy agents: implication in breast cancer treatment. Oncotarget (2011) 2(10):797–809. doi: 10.18632/oncotarget.343 22006582 PMC3248157

[22] Pantziarka P Bouche G Sukhatme V Meheus L Rooman I Sukhatme VP. Repurposing drugs in oncology (ReDO) - propranolol as an anti-cancer agent. ecancermedicalscience (2016) 12:10. doi: 10.3332/ecancer.2016.680 PMC5102691 27899953

[23] Pasquier E André N Street J Chougule A Rekhi B Ghosh J. Effective management of advanced angiosarcoma by the synergistic combination of propranolol and vinblastine-based metronomic chemotherapy: A bench to bedside study. EBioMedicine (2016) 6:87–95. doi: 10.1016/j.ebiom.2016.02.026 27211551 PMC4856748

[24] Saha J Kim JH Amaya CN Witcher C Khammanivong A Korpela DM. Propranolol sensitizes vascular sarcoma cells to doxorubicin by altering lysosomal drug sequestration and drug efflux. Front Oncol (2021) 1:10 doi: 10.3389/fonc.2020.614288 PMC7882688 33598432

[25] Fjæstad KY Rømer AMA Goitea V Johansen AZ Thorseth ML Carretta M. Blockade of beta-adrenergic receptors reduces cancer growth and enhances the response to anti-CTLA4 therapy by modulating the tumor microenvironment. Oncogene (2022) 41(9):1364–75. doi: 10.1038/s41388-021-02170-0 PMC8881216 35017664

[26] Sasaki M North PE Elsey J Bubley J Rao S Jung Y. Propranolol exhibits activity against hemangiomas independent of beta blockade. NPJ Precis Oncol (2019) 3(1):27. doi: 10.1038/s41698-019-0099-9 31701018 PMC6825155

[27] Amaya CN Perkins M Belmont A Herrera C Nasrazadani A Vargas A. Non-selective beta blockers inhibit angiosarcoma cell viability and increase progression free- and overall-survival in patients diagnosed with metastatic angiosarcoma. Oncoscience (2018) 5(3–4):109–19. doi: 10.18632/oncoscience.413 PMC5978448 29854879

[28] Luczynska E Rudnicki W Kargol J Szpor J Hodorowicz-Zaniewska D Wysocki PJ. Primary bilateral angiosarcoma of the breast treated with neoadjuvant chemotherapy combined with propranolol. Breast J (2021) 27(10):781–6. doi: 10.1111/tbj.14272 34263505

[29] Fiste O Dimos A Kardara VE Ballasis K Karampeazis A. Propranolol and weekly paclitaxel in the treatment of metastatic heart angiosarcoma. Cureus (2020) 12(12). doi: 10.7759/cureus.12262 PMC7834548 33520481

[30] Daguzé J Saint-Jean M Dréno B. Large Nose angiosarcoma treated effectively with oral cyclophosphamide combined with propranolol. J Eur Acad Dermatol Venereol (2018) 32(2):e52–4. doi: 10.1111/jdv.14528 28833588

[31] Daguzé J Saint-Jean M Peuvrel L Cassagnau E Quéreux G Khammari A. Visceral metastatic angiosarcoma treated effectively with oral cyclophosphamide combined with propranolol. JAAD Case Rep (2016) 2(6):497–9. doi: 10.1016/j.jdcr.2016.10.005 PMC5161779 28004027

[32] Banavali S Pasquier E Andre N. Targeted therapy with propranolol and metronomic chemotherapy combination: sustained complete response of a relapsing metastatic angiosarcoma. Ecancermedicalscience (2015) 8:9. doi: 10.3332/ecancer.2015.499 PMC4303616 25624880

[33] Pramanik R Gogia A Malik PS Gogi R. Metastatic primary angiosarcoma of the breast: Can we tame it the metronomic way. Indian J Med Paediatr Oncol (2017) 38(2):228–31. doi: 10.4103/ijmpo.ijmpo_156_16 PMC5582568 28900339

[34] Galván DC Ayyappan AP Bryan BA. Regression of primary cardiac angiosarcoma and metastatic nodules following propranolol as a single agent treatment. Oncoscience (2018) 5(9–10):264–8. doi: 10.18632/oncoscience.472 PMC6231448 30460329

[35] Chow W Amaya CN Rains S Chow M Dickerson EB Bryan BA. Growth attenuation of cutaneous angiosarcoma with propranolol-mediated β-blockade. JAMA Dermatol (2015) 151(11):1226–9. doi: 10.1001/jamadermatol.2015.2554 26375166

